# Oncological recurrence following pathological complete response after neoadjuvant treatment in patients with esophageal cancer — a retrospective cohort study

**DOI:** 10.1007/s00423-023-03100-2

**Published:** 2023-09-18

**Authors:** Julian Hipp, Jasmina Kuvendjiska, Hans Christian Hillebrecht, Stephan Herrmann, Sylvia Timme-Bronsert, Stefan Fichtner-Feigl, Jens Hoeppner, Markus K. Diener

**Affiliations:** 1 Department of General and Visceral Surgery, Medical Center - University of Freiburg, Hugstetter Str. 55, 79100 Freiburg, Germany; 2 Institute for Surgical Pathology, Medical Center - University of Freiburg, Faculty of Medicine - University of Freiburg, Hugstetter Str. 55, 79106 Freiburg, Germany; 3 Department of Surgery, University Medical Center Schleswig-Holstein, UKSH Campus Lübeck, Ratzeburger Allee 160, 23538 Lübeck, Germany

**Keywords:** Esophagogastric junction cancer, Esophageal cancer, Visceral surgery, Oncologic surgery, Pathological complete response, Multimodal treatment, Neoadjuvant treatment, Chemotherapy, Chemoradiation

## Abstract

**Background:**

To evaluate recurrence in patients with post-neoadjuvant pathological complete response (pCR) and in patients with complete response of primary tumor but persisting lymphatic spread of disease (non-pCR, ypT0ypN +) of esophageal cancer.

**Methods:**

Seventy-five patients (63 pCR, 12 non-pCR) were analyzed retrospectively. Pattern and incidence of local and distant recurrence as well as the impact on overall (OS) and disease-free survival (DFS) were evaluated. The efficacy of neoadjuvant chemotherapy according to FLOT protocol was compared to neoadjuvant chemoradiation according to CROSS protocol.

**Results:**

In the pCR group, isolated local recurrence was diagnosed in 3%, while no isolated local recurrence was observed in the non-pCR group due to the high incidence of distant recurrence. Distant recurrence was most common in both cohorts (isolated distant recurrence: pCR group 10% to non-pCR group 55%; simultaneous distant and local recurrence: pCR group 3% to non-pCR group 18%). Median time to distant recurrence was 5.5 months, and median time to local recurrence was 8.0 months. Cumulative incidence of distant recurrence (with and without simultaneous local recurrence) was 16% (± 6%) in pCR patients and 79% (± 13%) in non-pCR patients (hazard ratio (HR) 0.123) estimated by Kaplan–Meier method. OS (HR 0.231) and DFS (HR 0.226) were significantly improved in patients with pCR compared to patients with non-pCR. Advantages for FLOT protocol compared to CROSS protocol, especially with regard to distant control of disease (HR 0.278), were observed (OS (HR 0.361), DFS (HR 0.226)).

**Conclusion:**

Distant recurrence is the predominant site of treatment failure in patients with pCR and non-pCR grade 1a regression, whereby recurrence rates are much higher in patients with non-pCR.

**Supplementary Information:**

The online version contains supplementary material available at 10.1007/s00423-023-03100-2.

## Introduction

Globally, esophageal cancer (EC) is the 7th most common cancer and accounts for half a million deaths annually [1]. While the incidence of esophageal squamous cell cancer (ESCC) is stable, especially in western countries, the incidence of esophageal adenocarcinoma (EAC) and esophagogastric junction cancer (AEG) is on the rise [2]. Standard treatment options for locally advanced EC are neoadjuvant chemoradiation (nCRT) according to the CROSS protocol (EAC and ESCC) or perioperative/neoadjuvant chemotherapy (nCT) according to FLOT protocol (EAC) [3, 4]. This is typically followed by surgical resection of the tumor either by partial esophagectomy or transhiatal extended gastrectomy. A substantial fraction of patients achieves pathological complete response (pCR) with neoadjuvant treatment and therefore does not exhibit remaining vital tumor cells on postoperative histopathological examination. pCR is diagnosed in 16–35% of EAC patients after nCT and in 48% of ESCC patients after nCRT and correlates with improved overall (OS) and disease-free survival (DFS) [5, 6].

In recent years, the option of organ-preserving treatment by active surveillance and surgery as needed compared to standard surgery on principle in patients with clinical complete response (cCR) after neoadjuvant treatment has come into focus [7, 8]. Active surveillance offers the option of organ preservation, presumably without significantly increasing the rate of uncontrolled local recurrence/regrowth. The key for successful active surveillance seems to be accurate identification and classification of patients with cCR and conformity with pCR. At present, the prospectively evaluated preSANO protocol describes the most in-depth post-neoadjuvant work-up to identify patients with cCR. Comparison of cCR and pCR demonstrated a sensitivity of 74% and specificity of 77%. This leads to a negative predictive value of 45%, and especially, the accurate determination of the N stage is a difficult task [9]. However, active surveillance seems to have no significant impact on distant recurrence compared to current standard treatment options. The distant recurrence rate is mainly determined by the lymph node status of disease after neoadjuvant treatment [6, 8, 10]. Currently, several ongoing and planned trials are trying to generate evidence for future decision-making (SANO (NTR6803), ESOSTRATE (NCT02551458), and ESORES (preliminary registration identifier: DRKS 00022801)). Hence, the assessment of patients with “true” pCR (ypT0ypN0cM0) and those rare patients with complete response of the primary tumor but persisting lymphatic spread of disease (non-pCR, ypT0ypN + cM0) seems to be of utmost importance.

The main goals of our study were therefore to describe and compare the differences in the incidence and pattern of recurrence in patients with pCR and non-pCR, to compare the survival outcomes, and to compare the efficacy of both neoadjuvant treatment options with regard to local and distant control of tumor recurrence.

## Methods

### Patient selection

**Fig. 1:**
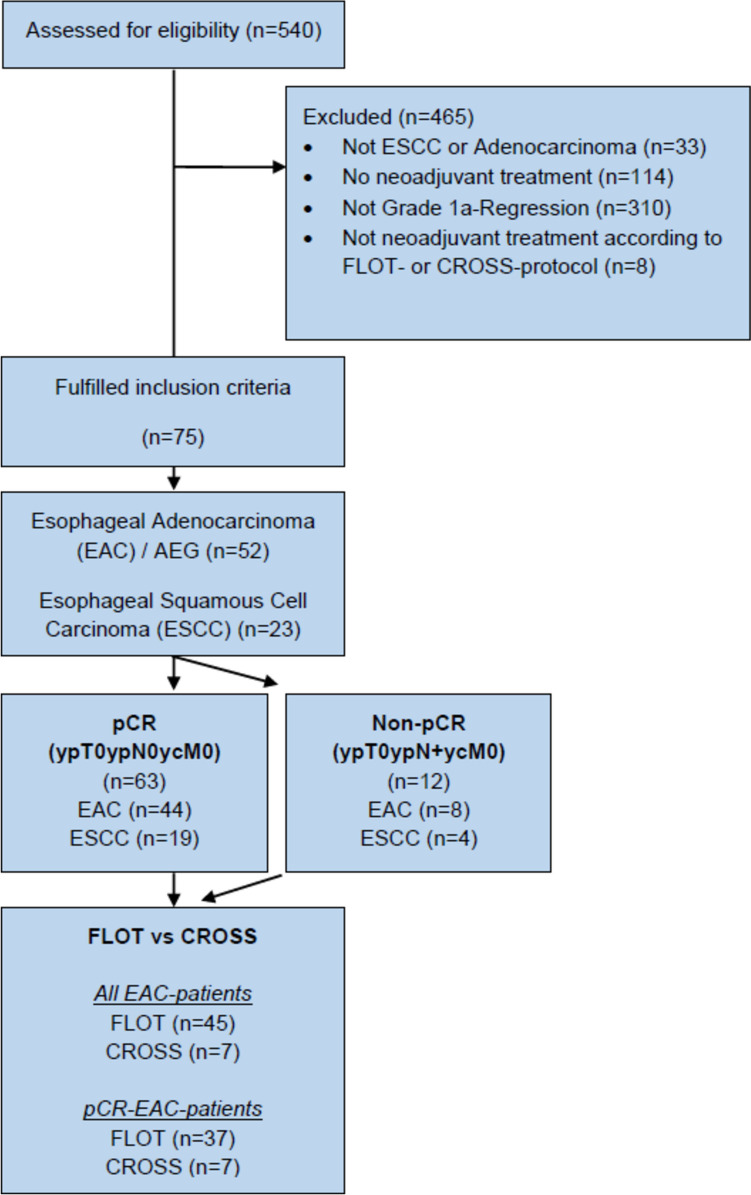
Study flow chart

This study is reported in accordance with the STROBE statement [11]. Patient data from 01/2014 to 01/2021 are based on our single-center database for upper gastrointestinal surgery (DRKS 00024369) and evaluated retrospectively. According to the study protocol, all consecutive patients with EC (ESCC and EAC), AEG, and adenocarcinoma with gastric location were screened for this study. Further inclusion criteria were the preoperative administration of either neoadjuvant chemotherapy according to the FLOT protocol [4] or neoadjuvant chemoradiation according to the CROSS protocol [3] and proof of postoperative pathological regression grade 1a (pathological complete response of primary tumor) according to Becker et al. [12] on postoperative histopathological examination. Out of 540 patients with upper gastrointestinal cancer, 393 patients with EC (341 with EAC, 52 with ESCC) were treated with neoadjuvant multimodal treatment in curative intent. Out of these patients, 75 (52 EAC, 23 ESCC) fulfilled all inclusion criteria and were included for all subsequent analyses (Fig. 1). The study was reviewed and approved by the Ethics Committee of the University of Freiburg (21–1093 and 21–1713).

The following variables were extracted from our prospectively maintained database: age, gender, BMI (kg/m^2^), the Royal College of Surgeons (RCS)-Charlson score [13], pretherapeutic uT stage, pretherapeutic uN stage, and pretherapeutic cM stage according to the American Joint Committee on Cancer/UICC 8th cancer staging manual [14] as well as the postoperative TNM classification, pretherapeutic grading, neoadjuvant treatment modality, the interval between end of neoadjuvant treatment and surgery, type of surgical procedure, and the comprehensive complication index (CCI) [15]. Due to the inclusion criteria, all patients exhibited a ypT0 stage and R0 resection status and had a regression grade 1a according to Becker et al. on postoperative histopathological examination [12]. Furthermore, all patients had a ycM0 situation. Based on negative post-neoadjuvant lymphatic spread, 63 patients (44 EAC and 19 ESCC) were classified as pathological complete response (pCR). Twelve patients (8 EAC, 4 ESCC) with evidence of post-neoadjuvant lymphatic spread (ypN1-3 (ypN +) classification) of disease were classified as non-pathological complete response (non-pCR).

### Statistical analysis

Oncologic follow-up was performed according to the German and NCCN guidelines for esophageal cancer treatment [16, 17]. Patients had follow-up computed tomography scans (CT) every 6 months for the first 2 years after surgery and then every 12 months. Based on development of symptoms suspicious for recurrence or post-treatment complications, out-of-routine CT scans as well as endoscopic controls were performed. A total of 42 patients had endoscopic controls. CT scans were available for 63 patients in our radiologic database (DeepUnity Diagnostic 1.1.0.1, Daedalus Healthcare, Bonn, Germany). The patients’ general practitioners or oncologists were contacted to complete the missing follow-up data; the most recent dated November 2022. Median follow-up for disease-free survival (DFS) data was 46 months as estimated by the reversed Kaplan–Meier method [18]. Time and location of recurrence (local, distant) were recorded. Disease-free survival was defined as the interval from surgery to detection of recurrent disease or death from any cause. Local treatment failure was defined as recurrence within the field of surgery. Local treatment failure was further subdivided into endoluminal recurrence, extraluminal/local lymphatic recurrence (supraclavicular recurrence, mediastinal recurrence, or lymphatic recurrence along the vessels of the coeliac trunk), or both. Distant treatment failure was subdivided into distant lymphatic recurrence (outside of the standard D2 lymphadenectomy and the above-mentioned locations), hepatic recurrence, pulmonary recurrence, peritoneal recurrence, recurrence as bone metastases, recurrence as brain metastases (or meningeal carcinomatosis), or recurrence in other sites (one patient with pleural carcinomatosis and one patient with adrenal metastasis). Cumulative incidence of isolated local recurrence and distant recurrence (with or without simultaneous local recurrence) were estimated by the Kaplan–Meier method. Overall survival (OS) data were systematically obtained via the local cancer registry of our Comprehensive Cancer Center Freiburg (CCCF), and follow-up data were collected until November 2022. OS was defined as the interval from surgery to death from any cause. Actuarial survival was calculated by univariate analysis using the Kaplan–Meier method, with log-rank testing for comparison of subgroups. Hazard ratios (HR) were estimated by univariate Cox proportional hazard models. The median follow-up for OS data was 48 months calculated with the reverse Kaplan–Meier method. Results are presented as mean (± standard deviation), median (interquartile range), or as number (percent). We used Mann–Whitney *U*-test for descriptive analysis for non-parametric variables and Pearson’s chi-squared test for categorical variables. Statistical analysis was performed using SPSS version 28.0.1.0 (IBM Corp., Armonk, NY, USA) and R version 4.0.0 (R Foundation for Statistical Computing, Vienna, Austria) with R Studio (R Studio Inc., Boston, MA, USA) and additional packages ggplot2 and survminer. Differences were considered statistically significant when *p* < 0.05.

## Results

Patient characteristics are outlined in Table 1. The median age was 62 years, 54 (72%) of the patients were male, 45 (60%) of patients received nCT according to the FLOT protocol, and 30 (40%) received nCRT according to the CROSS protocol. Treatment-associated 90-day mortality was 3/75 (4%) patients. For the purpose of this study, these patients were excluded from further disease-free survival and site of recurrence analyses, but not from OS analyses.

**Table 1:** Patient cohort

Variable		All patients (*n* = 75)	pCR (ypT0ypN0)		Non-pCR (ypT0ypN +)	
			EAC (*n* = 44)	ESCC (*n* = 19)	EAC (*n* = 8)	ESCC (*n* = 4)
Age (years) (*n* = 75)		62 (59–72)	63 (59–72)	62 (56–72)	60 (51–72)	69 (54–76)
Gender	Female	21 (28%)	14 (32%)	4 (21%)	2 (25%)	1 (25%)
	Male	54 (72%)	30 (68%)	15 (79%)	6 (75%)	3 (75%)
BMI (Kg/m^2^) (*n* = 75)		25.7 (23.7–28.4)	26.3 (23.8–28.7)	24.3 (22.5–26.6)	24.7 (23.9–28.6)	23.8 (22.1–28.3)
RCS Charlson Index	0	0 (0%)	0 (0%)	0 (0%)	0 (0%)	0 (0%)
	1	42 (56%)	24 (55%)	11 (58%)	4 (50%)	3 (75%)
	2	22 (29%)	14 (32%)	4 (21%)	4 (50%)	0 (0%)
	≥ 3	11 (15%)	6 (14%)	4 (21%)	0 (0%)	1 (25%)
uT stage	T0/Tis	1 (1%)	1 (2%)	0 (0%)	0 (0%)	0 (0%)
	T1	4 (5%)	1 (2%)	2 (11%)	0 (0%)	1 (25%)
	T2	11 (15%)	7 (16%)	3 (16%)	0 (0%)	1 (25%)
	T3	42 (56%)	28 (64%)	8 (42%)	5 (63%)	1 (25%)
	T4	8 (11%)	2 (5%)	3 (16%)	2 (25%)	1 (25%)
	NA	9 (12%)	5 (11%)	3 (16%)	1 (13%)	0 (0%)
uN stage	uN-	16 (21%)	10 (23%)	4 (22%)	2 (25%)	0 (0%)
	uN +	50 (67%)	29 (66%)	12 (63%)	5 (63%)	4 (100%)
	NA	9 (12%)	5 (11%)	3 (16%)	1 (13%)	0 (0%)
cM stage	cM0	66 (88%)	41 (93%)	19 (100%)	7 (88%)	1 (75%)
	cMX/1	9 (12%)	3 (7%)	0 (0%)	1 (13%)	1 (25%)
Grading	GX	31 (41%)	19 (43%)	8 (42%)	3 (38%)	1 (25%)
	G1	3 (4%)	2 (5%)	1 (5%)	0 (0%)	0 (0%)
	G2	23 (31%)	11 (25%)	7 (37%)	3 (38%)	2 (50%)
	G3	18 (24%)	12 (27%)	3 (16%)	2 (25%)	1 (25%)
Neoadjuvant chemotherapy according to FLOT protocol		45 (60%)	37 (84%)	0 (0%)	8 (100%)	0 (0%)
Neoadjuvant chemoradiation according to CROSS protocol		30 (40%)	7 (16%)	19 (100%)	0 (0%)	4 (100%)
Interval to surgery (end of neoadjuvant treatment to surgery) (days) (*n* = 67)		44 (37–60)	42 (35.5–52)	48.5 (38–61.5)	53.5 (21–88)	62.5 (60–84)
Surgical procedure	2-field esophagectomy	63 (84%)	34 (77%)	19 (100%)	6 (75%)	4 (100%)
	Transhiatal extended gastrectomy	2 (3%)	1 (2%)	0 (0%)	1 (13%)	0 (0%)
	Gastrectomy	7 (9%)	7 (16%)	0 (0%)	0 (0%)	0 (0%)
	Subtotal gastrectomy	2 (3%)	1 (2%)	0 (0%)	1 (13%)	0 (0%)
	Gastrectomy + HIPEC	1 (1%)	1 (2%)1	0 (0%)	0 (0%)	0 (0%)
CCI (*n* = 75)		22.6 (0–40.6)	23.4 (0–40.4)	20.9 (0–56.7)	29.2 (0–41.5)	35.7 (23.4–85.1)

### Pattern of recurrence

The details on the patterns of postoperative recurrence of EC after complete pathological response following neoadjuvant treatment are outlined in Table 2 and supplementary data table S1. Twenty-six events were observed in 18 patients; the recurrence rate was 16% (10/61) in patients with pCR and 73% (8/11) in non-pCR patients.

**Table 2:** Site of recurrence compared by post-neoadjuvant regression status

Site of failure		pCR (ypT0ypN0) (*n* = 61)	Non-pCR (ypT0ypN +) (*n* = 11)	*p*-value
Recurrence	No	51 (84%)	3 (27%)	< 0.001
	Yes	10 (16%)	8 (73%)	
Recurrence detail	No	51 (84%)	3 (27%)	< 0.001
	Isolated local recurrence	2 (3%)	0 (0%)	
	Isolated systemic recurrence	6 (10%)	6 (55%)	
	Local and systemic recurrence	2 (3%)	2 (18%)	
Total sites of failure		15 in 10 patients	11 in 8 patients	
Local failure	No	57 (93%)	9 (82%)	0.199
	Endoluminal recurrence	0 (0%)	0 (0%)	
	Local lymphatic metastases	4 (7%)	2 (18%)	
	• Supraclavicular lymph nodes	0 (0%)	0 (0%)	0.221
	• Mediastinal lymphatic recurrence	2 (50%)	0 (0%)	
	• Coeliac trunk	2 (50%)	2 (50%)	
Distant failure	Distant lymphatic	2/61 (3%)	2/11 (18%)	0.047
	Hepatic recurrence	1/61 (2%)	1/11 (9%)	0.166
	Pulmonary recurrence	5/61 (8%)	1/11 (9%)	0.921
	Peritoneal recurrence	1/61 (2%)	0/11 (0%)	0.669
	Bone metastases	1/61 (2%)	1/11 (9%)	0.166
	Brain metastases	0/61 (0%)	3/11 (27%)	< 0.001
	Other location	1/61 (2%)	1/11 (9%) (pleural carcinomatosis)	0.166

Recurrence rate was 12% (5/37) in patients with EAC and 26% (5/19) in patients with ESCC following pCR. Most cases of recurrence were distant metastases without local recurrence (pCR group: 6/61 (10%); non-pCR group: 6/11 (55%)). Isolated local recurrence of EC after grade 1a regression was a rare event in both cohorts. In the pCR group, isolated local recurrence was diagnosed in 2 patients (3%), while no isolated local recurrence was observed in the non-pCR group. All cases of local recurrence were local extraluminal/lymphatic recurrences in mediastinal lymph nodes or along the coeliac trunk. No endoluminal recurrences were observed.

Simultaneous distant and local recurrence was observed in 2/61 (3%) in the pCR group and in 2/11 (18%) in the non-pCR group. Regarding the location of distant recurrence, pulmonary recurrence was the most frequently observed site of recurrence in patients with pCR —especially in patients with ESCC (EAC: 1/42; ESCC: 4/19; *p* = 0.014), while significantly more distant lymphatic recurrences and intracerebral metastases were observed in the non-pCR group.

**Fig. 2:**
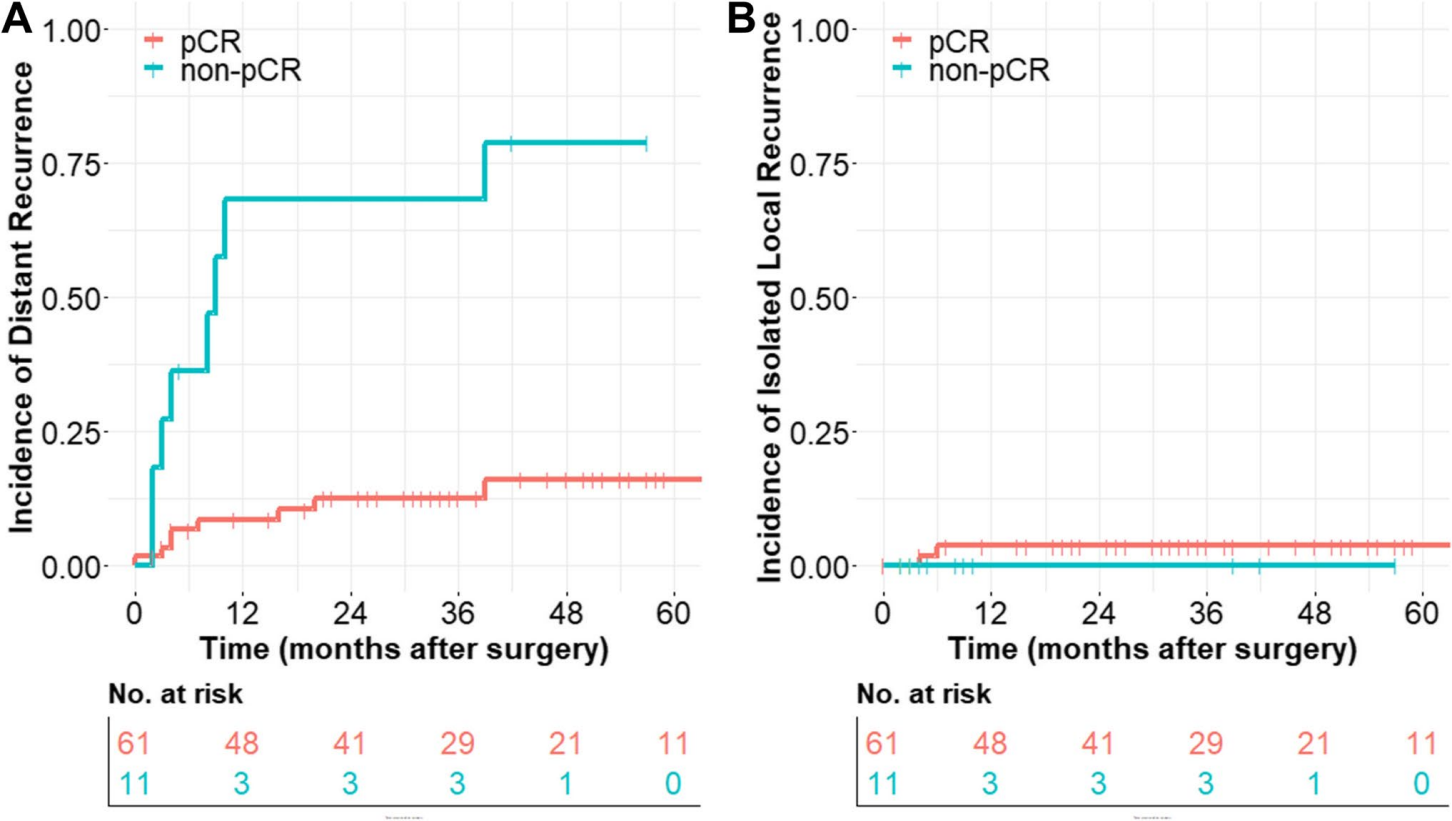
Timing of recurrence in patients with pCR and non-pCR after neoadjuvant treatment for esophageal cancer. **A** Distant recurrence: in patients with pCR, distant recurrence (with or without simultaneous local recurrence) occurred in 16% (± 6%) of patients, while in non-pCR patients, distant recurrence occurred in 79% (± 13%) of patients following R0 resection of esophageal cancer (HR 0.123 (95% CI: 0.046–0.331, *p* < 0.001). **B** Isolated local recurrence: isolated local recurrences occurred in 4% (± 2%) of patients with pCR. Isolated local recurrence of EC did not occur in non-pCR patients in our cohort due to the devastating incidence of simultaneous distant recurrence of disease, although 2 of 11 patients (18%) did have simultaneous local recurrence (Table 2)

Timing and pattern of recurrence are shown in the cumulative incidence analysis in Fig. 2. Median time to distant recurrence was 5.5 (3.0–14.5) months, and median time to local recurrence was 8.0 (3.75–21.75) months. In patients with pCR, distant recurrence (with or without simultaneous local recurrence) occurred in 16% (± 6%) of patients, while in non-pCR patients, distant recurrence occurred in 79% (± 13%) of patients following R0 resection of esophageal cancer (HR 0.123 (95% CI: 0.046–0.331, *p* < 0.001). Isolated local recurrence is a rare event that occurred in 4% (± 2%) of patients with pCR. In the non-pCR group, isolated local recurrence did not occur in our cohort due to the high incidence of simultaneous distant recurrence of disease. This result of the cumulative incidence analysis should not be misinterpreted; 2 of 11 patients (18%) did have distant and local recurrence simultaneously, as shown in Table 2.

### Survival analysis

OS and DFS were significantly improved in patients with pCR compared to patients with non-pCR. The same observation was made for EAC patients and ESCC patients separately as well (Supplementary data table S2, Figs. S1 and S2).

The EAC patients in our patient cohort received two different types of neoadjuvant treatment — either neoadjuvant/perioperative chemotherapy according to FLOT protocol or neoadjuvant chemoradiation according to CROSS protocol. Based on the underlying database, 45 of 276 (16.3%) patients had grade 1a regression, and 37 of 276 (13.4%) patients had pCR after neoadjuvant chemotherapy according to FLOT protocol. Seven of 45 (15.5%) patients had grade 1a regression and pCR after neoadjuvant chemoradiation according to CROS protocol. The efficacy for the induction of grade 1a regression (*p* = 0.89) and pCR (*p* = 0.69) did not significantly differ between the two neoadjuvant treatment protocols. Patients receiving CROSS showed a trend towards being older but did not display higher degree of comorbidities. On the other hand, patients receiving FLOT had a trend towards a higher uN stage and cM stage in the pre-neoadjuvant situation (Table 3). The following observations have to be interpreted in light of this inhomogeneity of the different cohorts. OS and DFS were significantly improved in patients with pCR after nCT according to FLOT protocol compared to nCRT according to CROSS protocol. An improved DFS and a trend towards a better OS following neoadjuvant treatment according to FLOT protocol were also observed in the entire cohort of EAC patients. However, patients with pCR and non-pCR in the FLOT group were compared to only pCR patients in the CROSS group, as all non-pCR patients with EAC were treated with FLOT. Following neoadjuvant treatment according to CROSS protocol, site of failure was more frequently distant recurrence in all EAC patients and in the pCR cohort as well (Table 3, Fig. S3, and Supplementary data tables S3 and S4). The cumulative incidence of distant recurrence (with and without simultaneous local recurrence) was 15% (± 6%) in patients after FLOT compared to 49% (± 20%) following nCRT according to CROSS protocol in all EAC patients (log-rank test: *p* = 0.050; HR 0.278 (95% CI: 0.071–1.098)). In the pCR cohort of EAC patients (Table 3), the cumulative incidence of distant recurrence (with and without simultaneous local recurrence) was 3% (± 3%) following neoadjuvant treatment according to FLOT protocol, while after neoadjuvant chemoradiation according to CROSS protocol, patients still had distant recurrence in the above-mentioned probability (log-rank test: *p* < 0.001, HR 0.041 (95% CI: 0.004–0.406)) (Supplementary data Fig. S4). Isolated local recurrence occurred in 0% of patients after FLOT and in 17% (± 15%) in both EAC cohorts cohort after nCRT (*p* = 0.010 and *p* = 0.017).

**Table 3:** Comparison of EAC pCR patients receiving neoadjuvant treatment according to FLOT and CROSS protocol

Variable		nCT according to FLOT protocol (*n* = 37)	nCRT according to CROSS protocol (*n* = 7)	*p*-value
Age (years) (*n* = 44)		62 (57–70)	72 (65–75)	0.067
Gender	Female	13 (35%)	1 (14%)	0.277
	Male	24 (65%)	6 (86%)	
BMI (Kg/m^2^) (*n* = 44)		26.6 (24.3–28.7)	23.6 (22.0–27.6)	0.067
RCS Charlson index	0	0 (0%)	0 (0%)	0.980
	1	20 (55%)	4 (57%)	
	2	12 (32%)	2 (28%)	
	≥ 3	5 (14%)	1 (14%)	
uT stage	T0–T2	7 (21%)	2 (40%)	0.336
	T3–T4	27 (79%)	3 (60%)	
uN stage	uN-	8 (24%)	2 (33%)	0.639
	uN +	25 (76%)	4 (67%)	
cM stage	cM0	34 (92%)	7 (100%)	0.435
	cMX/1	3 (8%)	0 (0%)	
Grading	GX	16 (43%)	3 (43%)	0.935
	G1	2 (5%)	0 (0%)	
	G2	9 (24%)	2 (29%)	
	G3	10 (27%)	2 (29%)	
Interval to surgery (end of neoadjuvant treatment to surgery) (days) (*n* = 37)		42 (35–51)	42 (38–64)	0.560
Surgical procedure	2-field esophagectomy	27 (73%)	7 (100%)	0.654
	Transhiatal extended Gastrectomy	1 (3%)	0 (0%)	
	Gastrectomy	7 (19%)	0 (0%)	
	Subtotal gastrectomy	1 (3%)	0 (0%)	
	Gastrectomy + HIPEC	1 (3%)	0 (0%)	
CCI (*n* = 44)		22.6 (0–43.4)	34.6 (0–39.7)	0.778
Site of recurrence	Isolated local recurrence	0 (0%)	1 (14%)	< 0.001
	Isolated distant recurrence	1 (3%)	1 (14%)	
	Distant and local recurrence	0 (0%)	2 (29%)	

## Discussion

In this study, we described the pattern of recurrence in patients with pCR (ypT0ypN0cM0) and non-pCR grade 1a regression (ypT0ypN + cM0) in patients with esophageal cancer after neoadjuvant therapy. Isolated local recurrence was a very rare event in both groups; hence, surgical resection of the primary tumor and systematic lymphadenectomy after neoadjuvant treatment achieves adequate local control of disease. Yet, distant recurrence of esophageal cancer was observed more frequently, in 16% of patients with pCR, and a staggering 79% of cases with non-pCR grade 1a regression.

The distant recurrence was distributed over several locations, with a slight cumulation of pulmonal and cerebral metastases. Patterns of recurrence differ between the two histopathological entities. Location and frequency of recurrence are in line with previous reports of recurrence in patients with pCR [19, 20].

Our study shows a detailed analysis of the recurrence pattern and incidence in patients with non-pCR grade 1a regression. The observed differences in the incidence of recurrence according to UICC stage 0 vs II/III also translated into survival differences in our entire cohort, as shown previously [6, 10, 21]. Due to recent advances in adjuvant treatment after nCRT and the establishment of immunotherapy for EC patients with non-pCR following the results of the CheckMate-577 trial, the survival advantages of pCR patients will probably be attenuated in future [22]. For the establishment of active surveillance protocols, endoluminal and lymph node status are to be assessed for the determination of local cCR. However, our results demonstrate that the main reason for non-pCR grade 1a regression is not inadequate local control of disease but rather distant recurrence. This translates into the overall prognosis of patients with pCR being mainly determined by rapid distant recurrence/progression of disease. Taking current sensitivity and specificity for the detection of persisting local disease based on the preSANO protocol into account, a relevant proportion of patients with non-pCR cannot be identified. Whether these patients profit from performing esophagectomy, which is unlikely to prevent early distant progression of disease, instead of prolonging chemotherapy or combining chemotherapy and immunotherapy has to be elucidated in future studies. With close observation intervals, local progression to irresectable regrowth is unlikely to occur in these patients, and prolonging chemotherapy or administration of immunotherapy is not delayed by surgical resection and possible complications. The diagnostic modalities and efforts to be made to identify lymph node status in patients with complete response of the primary tumor will have to be evaluated in the future, and, in line with other authors, we believe that future research should focus on the identification of biomolecular factors to improve prediction of regrowth or distant recurrence. This would help with the future stratification of patients in active surveillance concepts for clinical complete responders of EC [23, 24]. It is important to keep in mind that standard therapy of EC with pCR is still nCRT/nCT and surgical resection, until high-quality data from the above-mentioned RCTs are made available and present solid evidence for active surveillance. Till then, active surveillance should only be carried out within prospective studies.

Our study shows that in this small sub-cohort of patients with grade 1a regression and pCR from our large cohort of EC patients [6], nCT according to FLOT protocol seems to offer advantages compared to nCRT according to CROSS protocol with regard to overall and disease-free survival. This is especially due to superiority with regard to systemic control of disease. Neither of the two treatment options was superior with regard to induction of pCR in the first place in our study cohort. In contrast to this finding, a recent publication in *Annals of Surgery* showed that nCRT according to CROSS protocol was associated with a higher rate of pCR (18% vs. 10%) and more R0 resections than nCT according to FLOT protocol, but the postoperative 90-day mortality was higher as well (5% vs 1%), which is mainly attributed to postoperative pulmonary and cardiovascular complications [25]. Data on the comparison of survival differences of the two treatment options from high-quality retrospective studies are rare; results from randomized controlled trials are not yet available. Recent retrospective studies found no survival difference between FLOT and CROSS protocols but better post-neoadjuvant regression after nCRT [26, 27]. To our knowledge, there is a complete lack of data on the comparison of the efficacy of the two treatment options in patients with pCR. Hence, based on the current evidence, no clear recommendation for one of the two neoadjuvant treatment options can be made [28, 29]. Hopefully, with the upcoming results of the ESOPEC trial (NCT02509286), clinicians will have an evidence-based foundation for future clinical decision-making [30]. Possible advantages of nCRT according to CROSS protocol are therefore a higher probability of local tumor control and possibly pCR rate, while the main advantage of perioperative chemotherapy according to FLOT protocol is a better systemic control of disease. It will be interesting to see whether a combination of neoadjuvant radiotherapy and perioperative chemotherapy according to FLOT protocol will combine the above-mentioned possible advantages of both treatment options. This regimen is currently under investigation in the randomized RACE trial (NCT04375605) [31].

Limitations of our study include the retrospective nature as well as the single-center design of the study. Furthermore, the low number of cases, especially in the non-pCR cohort and the CROSS cohort, limits the generalizability of our results. However, we utilize a homogeneous cohort with only two distinct neoadjuvant protocols, either nCT or nCRT, leading to better comparability of treatment groups.

In conclusion, distant recurrence is the predominant site of treatment failure in patients with both pCR and non-pCR grade 1a regression, although recurrence rates are much higher in patients with non-pCR grade 1a regression. Future studies should focus on the improvement of distant control of disease in this cohort of patients.

### Supplementary Information

Below is the link to the electronic supplementary material.

Supplementary file1 (DOCX 3236 KB)

Supplementary file2 (PDF 212 KB)

## Data Availability

The datasets and codes generated and analyzed during the current study are available from the corresponding author upon reasonable request.
