# Supplementary material for: Oncological recurrence following pathological complete response after neoadjuvant treatment in patients with esophageal cancer — a retrospective cohort study
Source: Langenbecks Arch Surg. 2023 Sep 18;408(1):363. doi: 10.1007/s00423-023-03100-2 (PMC10506930; doi:10.1007/s00423-023-03100-2)
Supplement: Supplementary file 1 — Supplementary file1 (DOCX 3236 KB) [file 423_2023_3100_MOESM1_ESM.docx]

**Oncological recurrence following pathological complete response after neoadjuvant treatment in patients with esophageal cancer – a retrospective cohort study**

Julian Hipp, Jasmina Kuvendjiska, Hans Christian Hillebrecht, Stephan Herrmann, Sylvia Timme-Bronsert, Stefan Fichtner-Feigl, Jens Hoeppner and Markus K. Diener

**Fig. S1:** Survival of patients with pCR and non-pCR.

A) OS (p<0.001, HR 0.231 (95%-CI: 0.096-0.555)) and B) DFS (p<0.001, HR 0.226 (95%-CI: 0.090-0.568)) were significantly improved in patients with pCR compared to patients with non-pCR.


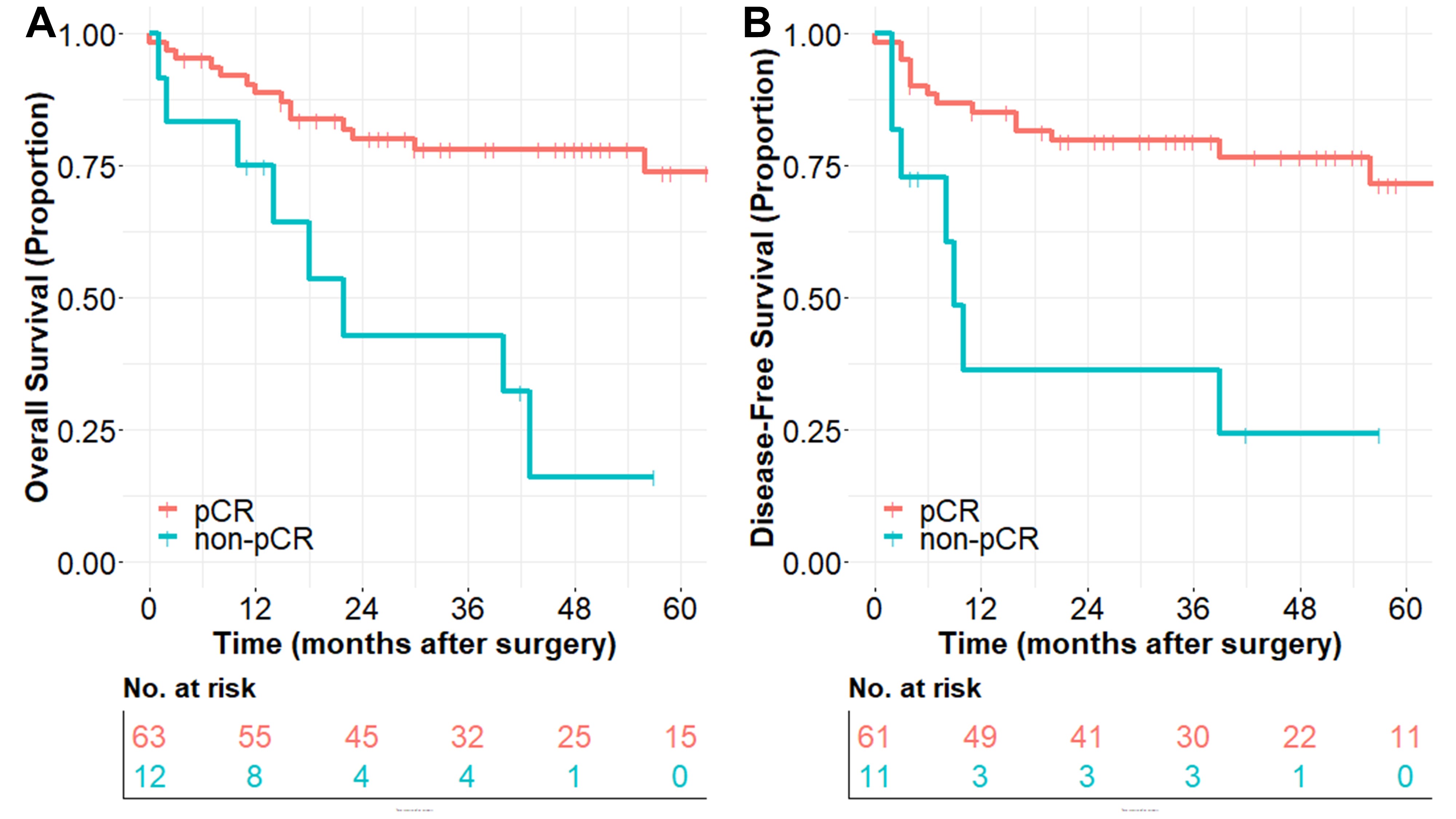


**Fig. S2:** Survival of EC patients with pCR compared to non-pCR in A) (p=0.002) and B) (p<0.001) EAC-patients and C) (p=0.035) and D) (p=0.331) ESCC-patients.


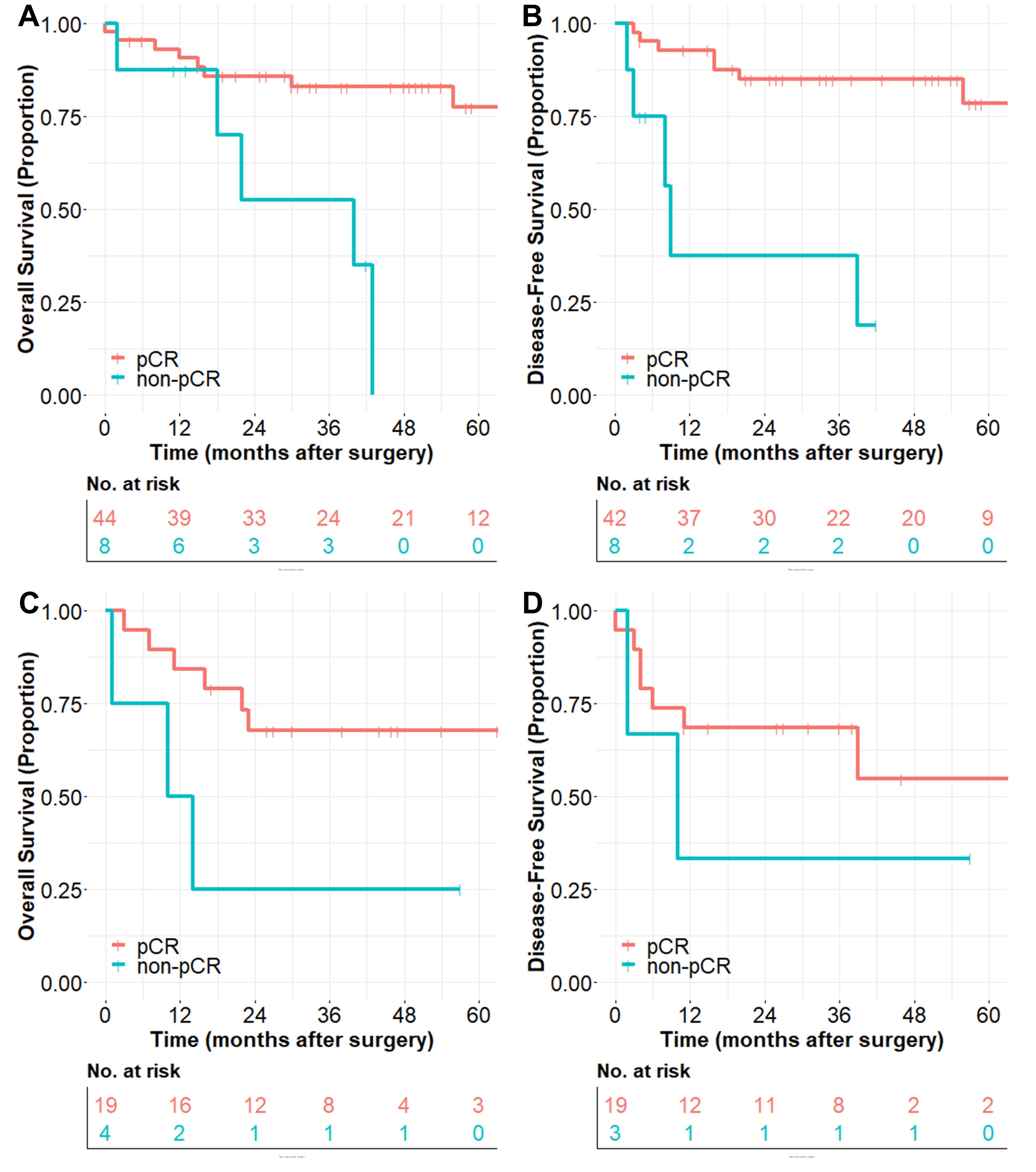


**Fig. S3:** Survival of EAC-patients according to neoadjuvant treatment modality.

A) and B) An improved DFS (p=0.009, HR 0.226 (95%-CI: 0.067-0.765)) and a trend towards a better OS (0.112, HR 0.361 (95%-CI: 0.097-1.339)) following neoadjuvant treatment according to FLOT-protocol was observed in the entire cohort of EAC-patients.

C) and D) OS (p=0.036, HR 0.237 (95%-CI: 0.055-1.017)) and DFS (p<0.001, HR 0.091 (95%-CI: 0.020-0.419)) was significantly improved in patients with pCR after nCT according to FLOT-protocol compared to nCRT according to CROSS-protocol.


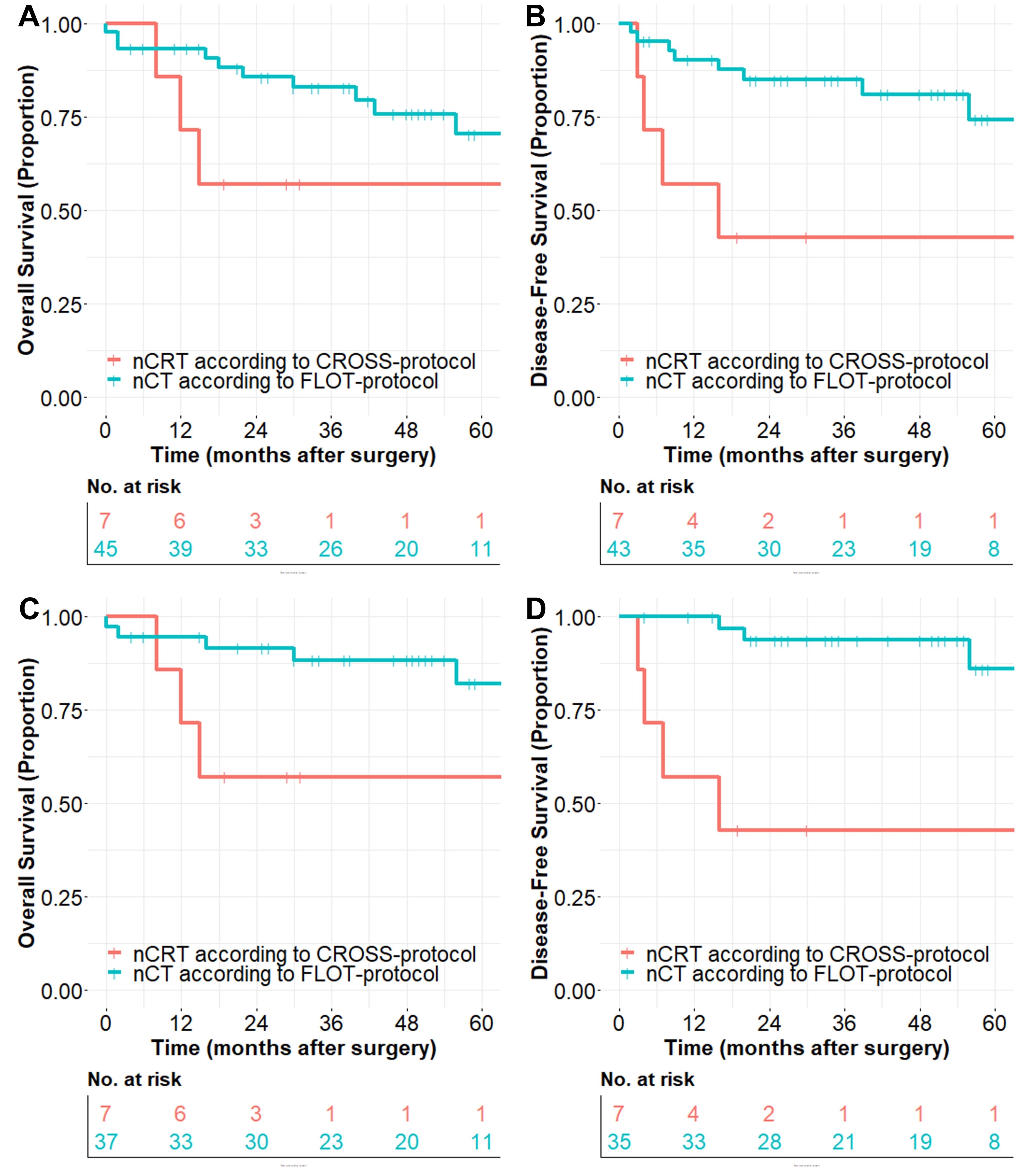


**Fig. S4:** Cumulative incidence of distant recurrence in EAC-patients according to neoadjuvant treatment modality.

A) The cumulative incidence of distant recurrence (with and without simultaneous local recurrence) was 15% (±6%) in patients after FLOT compared to 49% (±20%) in all EAC-patients (log rank-test: p=0.050; HR 0.278 (95%-CI: 0.071-1.098)).

B) In the pCR-cohort of EAC-patients, the cumulative incidence of distant recurrence (with and without simultaneous local recurrence) was 3% (±3%) following neoadjuvant treatment according to FLOT-protocol, while patients after neoadjuvant chemoradiation according to CROSS-protocol still had distant recurrence in the above mentioned probability (log rank-test: p<0.001, HR 0.041 (95%-CI: 0.004-0.406)).


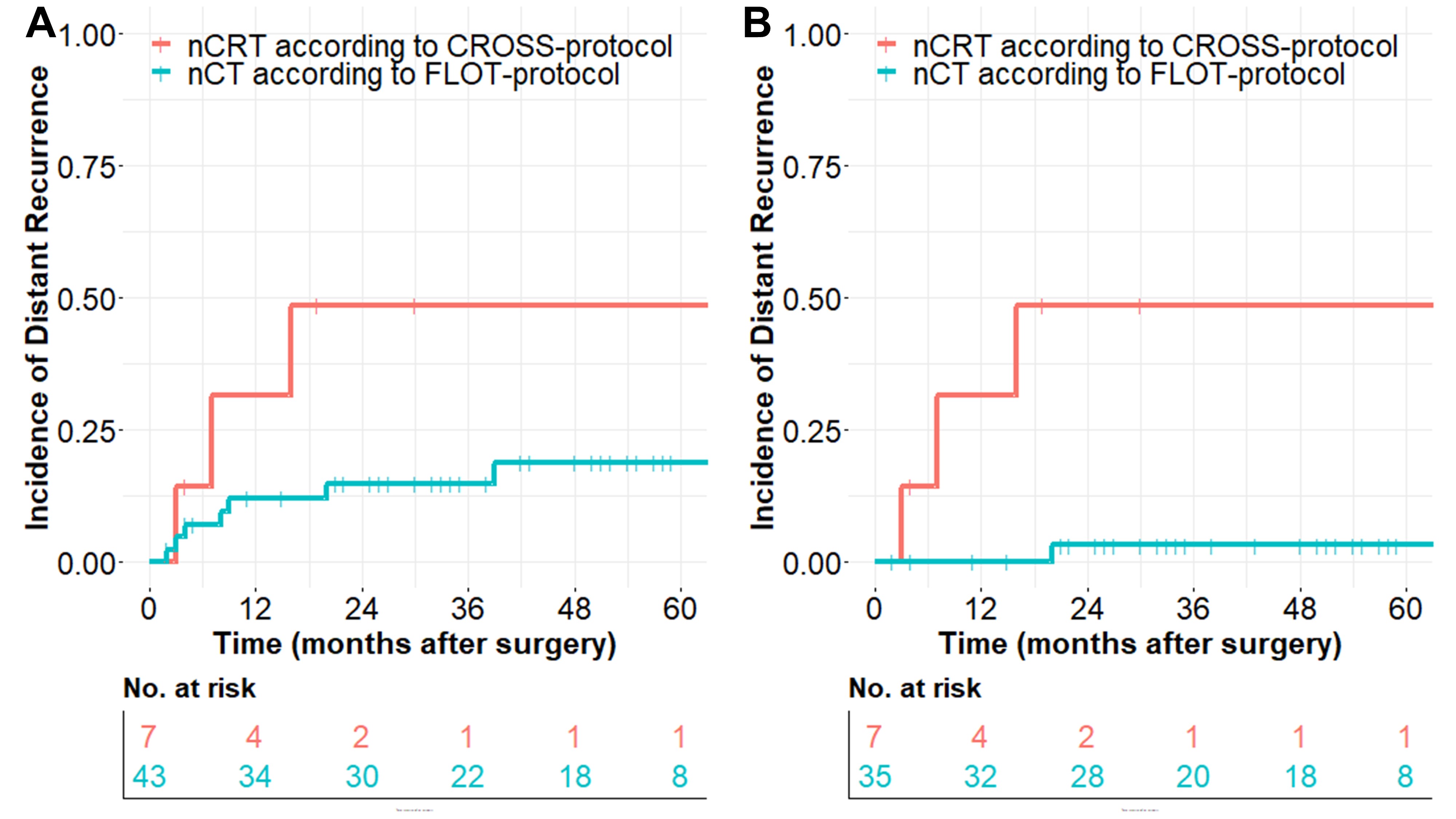


**Table S1:** Site of recurrence

| **Site of failure** | | **pCR (ypT0ypN0)** | | **p-value** | **Non-pCR (ypT0ypN+)** | | **p-value** |
| --- | --- | --- | --- | --- | --- | --- | --- |
|  |  | **EAC (n=42)** | **ESCC (n=19)** |  | **EAC (n=8)** | **ESCC (n=3)** |  |
| **Recurrence** | No Recurrence | 37 (88%) | 14 (74%) | 0.165 | 2 (25%) | 1 (33%) | 0.632 |
|  | Isolated Local Recurrence | 1 (2%) | 1 (5%) |  | 0 (0%) | 0 (0%) |  |
|  | Isolated Distant Recurrence | 2 (5%) | 4 (21%) |  | 5 (63%) | 1 (33%) |  |
|  | Local and systemic recurrence | 2 (5%) | 0 (0%) |  | 1 (13%) | 1 (33%) |  |
| **Total sites of failure** | | 8 in 5 patients | 7 in 5 patients |  | 7 in 6 patients | 4 in 2 patients |  |
| **Local failure** | No | 39 (93%) | 18 (95%) | 0.784 | 7 (88%) | 2 (67%) | 0.425 |
|  | Endoluminal recurrence | 0 (0%) | 0 (0%) |  | 0 (0%) | 0 (0%) |  |
|  | Local lymphatic metastases | 3 (7%) | 1 (5%) |  | 1 (13%) | 1 (33%) |  |
|  | - Supraclavicular lymphnodes | 0 (0%) | 0 (0%) | 0.248 | 0 (0%) | 0 (0%) | NA |
|  | - mediastinal lymphatic recurrence | 1 (33%) | 1 (100%) |  | 0 (0%) | 0 (0%) |  |
|  | - Coeliac trunk | 2 (66%) | 0 (0%) |  | 1 (100%) | 1 (100%) |  |
| **Distant failure** | Distant lymphatic | 1/42 (2%) | 1/19 (5%) | 0.558 | 1/8 (13%) | 1/3 (33%) | 0.425 |
|  | Hepatic recurrence | 1/42 (2%) | 0/19 (0%) | 0.498 | 0/8 (0%) | 1/3 (33%) | 0.087 |
|  | Pulmonary recurrence | 1/42 (2%) | 4/19 (21%) | 0.014 | 1/8 (13%) | 0/3 (0%) | 0.521 |
|  | Peritoneal recurrence | 1/42 (2%) | 0/19 (0%) | 0.498 | 0/8 (0%) | 0/3 (0%) | NA |
|  | Bone metastases | 1/42 (2%) | 0/19 (0%) | 0.498 | 1/8 (13%) | 0/3 (0%) | 0.521 |
|  | Brain metastases | 0/42 (0%) | 0/19 (0%) | NA | 3/8 (38%) | 0/3 (0%) | 0.214 |
|  | Other location | 0/42 (0%) | 1/19 (5%)* | 0.134 | 0/8 (0%) | 1/3 (33%) ^#^ | 0.087 |

*****Adrenal metastasis; ^#^Pleuracarcinomatosis

**Table S2:** Survival analyses

|  | **pCR** | **Non-pCR** |  |  |
| --- | --- | --- | --- | --- |
|  | **OS (months)** | **OS (months)** | **p-value** | **Hazard Ratio** |
| **All patients** | Median OS not reached | 22.0 (95%-CI: 10.5-33.5) months | p<0.001 | HR 0.231 (95%-CI: 0.096-0.555) |
| **EAC-patients** | Median OS not reached | 40.0 (95%-CI: 15.7-64.3) | p=0.002 | HR 0.191 (95%-CI: 0.060-0.611) |
| **ESCC-patients** | Median OS not reached | 10.0 (95%-CI: 0-22.7) | p=0.035 | HR 0.246 (95%-CI: 0.060-1.008) |
|  | **DFS (months)** | **DFS (months)** | **p-value** | **Hazard Ratio** |
| **All patients** | Median-DFS not reached | 9 (95%-CI: 6.3-11.7) | p<0.001 | HR 0.226 (95%-CI: 0.090-0.568) |
| **EAC-patients** | Median DFS not reached | 9.0 (95%-CI: 6.9-11.1) | p<0.001 | HR 0.123 (95%-CI: 0.036-0.414) |
| **ESCC-patients** | Median DFS not reached | 10.0 (95%-CI: 0-22.8) | p=0.331 | HR 0.467 (95%-CI: 0.096-2.268) |

**Table S3:** Survival analyses of EAC-patients undergoing neoadjuvant treatment according to FLOT- vs. CROSS-protocol.

|  | **FLOT-protocol** | **CROSS-protocol** |  |  |
| --- | --- | --- | --- | --- |
|  | **OS (months)** | **OS (months)** | **p-value** | **Hazard Ratio** |
| **All EAC-patients** | Median OS not reached | Median OS not reached | 0.112 | HR 0.361 (95%-CI: 0.097-1.339) |
| **pCR-EAC-patients** | Median OS not reached | Median OS not reached | 0.036 | HR 0.237 (95%-CI: 0.055-1.017) |
|  | **DFS (months)** | **DFS (months)** | **p-value** | **Hazard Ratio** |
| **All EAC-patients** | Median DFS not reached | 16.0 (95%-CI: 0-39.1) | 0.009 | HR 0.226 (95%-CI: 0.067-0.765), |
| **pCR-EAC-patients** | Median DFS not reached | 16.0 (95%-CI: 0-39.1) | <0.001 | HR 0.091 (95%-CI: 0.020-0.419) |

**Table S4:** Comparison of site of treatment failure in all EAC-patients.

|  | | | **nCT according to FLOT-protocol**  **(n=43)** | **nCRT according to CROSS-protocol**  **(n=7)** | **p-value** |
| --- | --- | --- | --- | --- | --- |
| **Site of recurrence** | **Isolated Local Recurrence** | 0 (0%) | | 1 (14%) | 0.002 |
|  | **Isolated Distant Recurrence** | 6 (14%) | | 1 (14%) |  |
|  | **Distant and Local Recurrence** | 1 (3%) | | 2 (29%) |  |
